# Sappanone A alleviates hypoxia/reoxygenation-induced cardiomyocytes injury through inhibition of mitochondrial apoptosis and activation of PI3K–Akt–Gsk-3β pathway

**DOI:** 10.1042/BSR20192442

**Published:** 2020-02-25

**Authors:** Xiaojing Shi, Guizhou Tao, Lili Ji, Ge Tian

**Affiliations:** Department of Cardiology, The First Affiliated Hospital of Jinzhou Medical University, Jinzhou, China

**Keywords:** Apoptosis, Mitochondrial apoptosis pathway, Myocardial ischemia reperfusion injury, PI3K-Akt-Gsk-3β pathway, Sappanone A

## Abstract

Myocardial ischemia reperfusion injury (MIRI) is a complex pathophysiological process involved with the activation of oxidative stress, inflammation and apoptosis. Sappanone A (SA), a homoisoflavanone isolated from the heartwood of *Caesalpinia sappan L.*, could exhibit antioxidant, anti-inflammatory and anti-apoptotic activities. Therefore, we assumed that SA has a potential use for preventing against MIRI. The present study aimed to investigate the effect of SA treatment on MIRI and its mechanism. Cardiomyocytes (H9c2 cells) were treated with SA for 1 h, followed by 6 h of hypoxia/3 h of reoxygenation. Cell viability assay was detected by CCK-8 assay. Apoptosis was measured by flow cytometry and Hoechst staining. Mitochondrial permeability transition pore (mPTP) opening and mitochondrial transmembrane potential (Δ*Ψ*m) were measured by spectrophotometry and JC-1 staining. The changes of mitochondrial apoptosis-related proteins and PI3K–Akt–Gsk-3β signaling pathway were evaluated by Western blotting. The results showed that SA pretreatment enhanced the cell viability and decreased the activity of myocardial enzyme in a dose-dependent manner. Moreover, SA pretreatment significantly inhibited apoptosis, blocked mPTP opening, suppressed the release of Δ*Ψ*m, prevented the cytochrome *c* releasing from mitochondria into cytoplasm, and repressed the cleavage of caspase-9 and caspase-3. Furthermore, SA pretreatment increased the phosphorylation levels of Akt and Gsk-3β but not of Stat-3. Meanwhile, the protective effect of SA was abrogated by PI3K inhibitor (LY294002). In conclusion, our results demonstrate that SA could prevent hypoxia/reoxygenation-induced cardiomyocytes injury through inhibition of mitochondrial apoptosis and activation of PI3K–Akt–Gsk-3β pathway. Thus, SA may have a potential use for the prevention of MIRI.

## Introduction

Ischemic heart disease is a leading cause of morbidity and mortality worldwide [1]. Acute myocardial infarction results from a complete occlusion of a coronary artery, usually caused by a superimposed thrombus on top of a ruptured atherosclerotic plaque [2]. Restoration of blood supply, namely reperfusion therapy is considered as the optimal way to rescue endangered myocardium. However, reperfusion sometimes itself may abnormally aggravate myocardial damage in clinical practice, a phenomenon known as myocardial ischemia reperfusion injury (MIRI) [3]. Growing evidence has demonstrated that several critical factors, such as oxidative stress, inflammation and apoptosis, mediate the detrimental effects of MIRI [4]. Therefore, inhibition of these mediators provides new therapeutic strategies against MIRI.

Sappanone A (SA) (chemical formula: C_16_H_12_O_5_), 4H-1-Benzopyran-4-one,3-[(3,4-dihydroxyphenyl)methylene]-2,3-dihydro-7-hydroxy-(9CI), a kind of homoisoflavanone, is originally isolated from the heartwood of *Caesalpinia sappan L.* [5]*.* Recent studies have reported that SA could exert multiple pharmacological effects, including antioxidant [6], anti-inflammatory [7] and anti-apoptotic [8] activities. For instance, SA treatment increased the level of Nrf2 and HO-1, decreased cisplatin-induced TNF-α and IL-1β production, and repressed NF-κB activation in a dose-dependent manner in human proximal tubule epithelial cells [6]. More interesting, SA pretreatment protected PC-12 cells against hypoxia-induced damage through inhibiting apoptosis [8]. However, whether SA treatment could prevent MIRI is still unknown. Considering SA possesses powerful antioxidant, anti-inflammatory and anti-apoptotic activities, we assumed that SA has a potential use for preventing against MIRI.

In the present study, we aimed to investigate the effect of SA treatment on MIRI and its mechanism in this process. Our results indicated that SA pretreatment protected cardiomyocytes against hypoxia/reoxygenation (H/R)-induced injury in a dose-dependent manner *in vitro*. The cardioprotection of SA was involved with the inhibition of mitochondrial apoptosis and activation of PI3K–Akt–Gsk-3β pathway.

## Materials and methods

### Cell culture and treatment

Rat cardiomyocytes (H9c2 cells) were purchased from Shanghai Institutes for Biological Sciences (Shanghai, China) and cultivated in Dulbecco’s Modified Eagle Medium contained 10% fetal bovine serum (FBS) under 37°C with 5% CO_2_ condition. To establish cardiomyocytes hypoxia/reoxygenation (H/R) model, H9c2 cells were transferred to Earle’s medium without glucose and FBS and cultivated in a hypoxic condition (90% N_2_, 5% CO_2_ and 5% O_2_ in a tri-gas incubator) for 6 h, and then Earle’s medium was removed and the cells were further cultivated with normal medium in an incubator with 5% CO_2_ at 37°C for 3 h of reoxygenation.

SA (CAS No. 102067-84-5, Purity ≥ 98%) was purchased form ChemFaces (Wuhan, China). SA was dissolved in DMSO (Sigma-Aldrich, St. Louis, MO, U.S.A.), and was diluted by culture medium until the concentration of DMSO less than 0.1%. H9c2 cells were treated by various concentration of SA for 1 h before hypoxia. About 10 μM LY294002 was added to culture medium 1 h prior to SA treatment to inhibit the activation of PI3K-Akt.

### Cell viability assay

The cells were seeded into 96-well plate (3000 cells per well) and the CCK-8 assay (KeyGEN Biotech, Nanjing, China) was performed to detect cell viability following to the manufacturer’s protocols.

### Myocardial enzyme test

When cardiomyocytes suffer from acute damage, lactate dehydrogenase (LDH), creatine kinase-MB (CK-MB) and Troponin (cTnI) will release into the culture medium. The activities of LDH and CK-MB, as well as the concentration of cTnI were detected using the LDH Assay Kit, CK-MB Assay Kit and Troponin Assay Kit (Jiancheng Biotech Co., Ltd, Nanjing, China) following to the manufacturer’s instructions.

### Apoptosis assay

To detect cell apoptosis *in vitro*, flow cytometry was carried out using the Annexin V-Fluorescein Isothiocyanate (FITC)/Propidium Iodide (PI) Kit (Dojindo, Kumamoto, Japan) following to the manufacturer’s recommendations.

### Hoechst staining assay

Cells were cultured in 24-well plates that were plated with glass slides in advance and treated with SA followed by H/R as previously described. After that cells were fixed with 4% paraformaldehyde for 30 min, and then incubated with 0.5 ml Hoechst 33342 solution (Wanlei, Shenyang, China) for 5 min at room temperature. After that cells were washed twice with PBS and the changes in nuclear morphology were observed under fluorescence microscopy with 350 nm excitation and 461 nm emission. The number of Hoechst-positive nuclei per optical field (at least 5 fields) was counted in three independent experiments and further calculated the ratio of apoptotic cell.

### Sensitivity of mitochondrial permeability transition pore (mPTP) to calcium ion (Ca^2+^)

The sensitivity of mPTP to Ca^2+^ could reflect the opening of mPTP [9]. The mitochondria of SA-treated cardiomyocytes were extracted from the cell lysates using Cell Mitochondria Isolation Kit (Beyotime) following to the manufacturer’s recommendations. The reaction of mPTP to Ca^2+^ was detected using Purified Mitochondrial Membrane Pore Channel Colorimetric Assay kit (GENMED, Shanghai, China) following to the manufacturer’s recommendations.

### Change of the mitochondrial transmembrane potential (Δ*Ψ*m)

mPTP opening can induce depolarization of the Δ*Ψ*m, and loss of Δ*ψ*m is considered to be an early event in the apoptotic process [10]. The change of Δ*Ψ*m was detected by using Mitochondrial Membrane Potential Assay Kit with JC-1 (Beyotime Institute of Biotechnology, Shanghai, China) according to the manufacturer’s protocol. JC-1 is an ideal fluorescent probe widely used for the detection of Δ*Ψ*m. When Δ*Ψ*m is normal, JC-1 accumulates in the matrix of mitochondria and forms J-aggregates that produce red fluorescence. Once Δ*Ψ*m is lost, JC-1 cannot aggregate in the matrix of the mitochondria and maintains a monomer that produces green fluorescence. The fluorescence was observed under a fluorescence microscope.

### Western blotting

Proteins were extracted from cell lysates using RIPA lysis Buffer and the protein concentration was measured using the Enhanced BCA Protein Assay Kit (Beyotime) following to the manufacturer’s recommendations. Proteins were denatured by heat, and then separated by SDS-PAGE electrophoresis, finally transferred to PVDF membranes. The membranes were blocked using 1% bovine serum albumin solution for 1 h at room temperature, and then incubated with primary antibodies, including anti-cytochrome *c* (1:1000; Abcam), anti-cleaved caspase-9 (1:1000; Abcam), anti-cleaved caspase-3 (1:1000; Abcam), anti-phospho-Akt (1:1000; Abcam), anti-Akt (1:1000; Abcam), anti-phospho-Gsk-3β (1:1000; Abcam), anti-Gsk-3β (1:1000; Abcam), anti-phospho-Stat-3 (1:1000; Abcam), anti-Stat-3 (1:1000; Abcam) anti-COXIV (1:1000; Abcam) and anti-β-actin (1:1000; Zhongshan Jinqiao Biotechnology, Beijing, China) at 4°C overnight, followed by incubation with HRP-conjugated secondary antibody (1:5000; Zhongshan Jinqiao Biotechnology, Beijing, China) at room temperature for 30 min. Protein bands were detected using a BeyoECL Plus Kit (Beyotime) following to the manufacturer’s protocols. Relative densitometry was analyzed using Image J2x analysis software (NIH, U.S.A.).

### Statistical analysis

Data were expressed as the mean ± standard deviation (SD). To analyze the differences between two groups, Student’s *t* test was conducted, and if analyzing the differences between multiple groups, one-way analysis of variance was used. *P* < 0.05 indicated a statistically significant difference. All statistical analyses were carried out using SPSS version 17.0 software (SPSS Inc., Chicago, IL).

## Results

### SA pretreatment protected cardiomyocytes against H/R-induced injury in a dose-dependent manner

**Figure 1:**
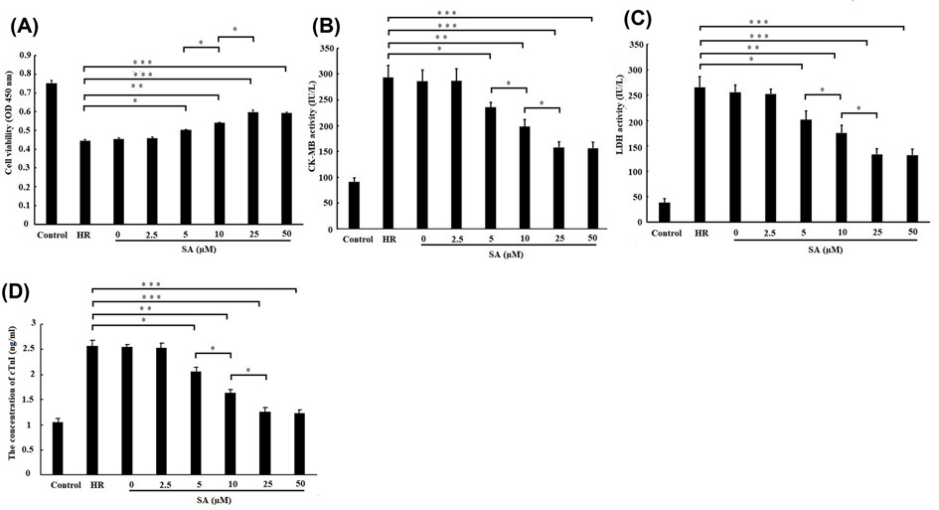
Sappanone A (SA) pretreatment protected cardiomyocytes against H/R-induced injury in a dose-dependent manner H9c2 cardiomyocytes were treated by various dose of SA for 1 h, followed by 6 h of hypoxia/3 h of reoxygenation. (**A**) Cell viability was detected by CCK-8 assay. (**B**) The creatine kinase-MB (CK-MB) and (**C**) lactate dehydrogenase (LDH) activity in culture medium were measured by spectrophotometry. (**D**) The concentration of Troponin (cTnI) in culture medium was measured by spectrophotometry. Data are presented as the mean ± standard deviation from three independent experiments. * *P* < 0.05; ** *P* < 0.01; *** *P* < 0.001.

The cell viability was evaluated after treated with various doses of SA (0, 2.5, 5, 10, 25 and 50 µM) using CCK-8 assay. As shown in Figure 1A, the cell viability was enhanced by 5, 10, 25 and 50 µM SA pretreatment. Moreover, cell viability in 10 µM SA-treated cells was higher than 5 µM SA-treated cells. Meanwhile, cell viability in 25 µM SA-treated cells was further higher than 10 µM SA-treated cells. However, there is no difference in cell viability between 25 and 50 µM SA-treated cells. The activities of CK-MB and LDH released from cells, as well as the concentration of cTnIalso indicated the similar results to cell viability (Figure 1B–D). Taken together, these results suggested that SA protected cardiomyocytes against H/R-induced injury in a dose-dependent manner, and 25 µM SA was determined to use for further investigation due to its optimal cardioprotection.

### SA pretreatment inhibited H/R induced apoptosis

**Figure 2:**
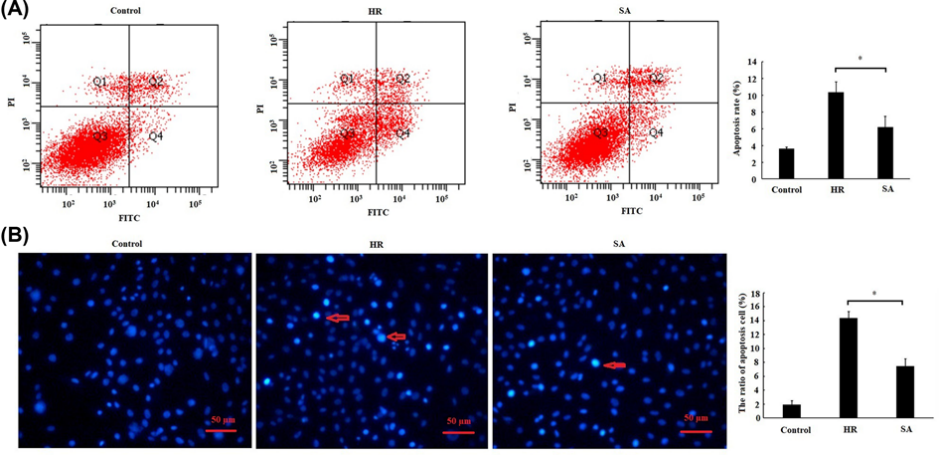
Sappanone A (SA) pretreatment inhibited H/R-induced apoptosis H9c2 cardiomyocytes were treated by 25 µM SA for 1 h, followed by 6 h of hypoxia/3 h of reoxygenation. (**A**) Cell apoptosis was detected by flow cytometry. Cells in the lower right quadrant represent apoptosis cells. (**B**) Hoechst staining assay was performed to assess cell apoptosis in H9c2 cells. Hoechst-positive nuclei (apoptotic nuclei) showed dense and high-density fluorescence, as indicated by arrows, and the percentage of Hoechst-positive nuclei per optical field (at least 5 fields) was counted. Magnification: ×400, scale bars: 50 μm. Data are presented as the mean ± standard deviation from three independent experiments; * *P* < 0.05.

The result of flow cytometry indicated that the apoptosis rate was significantly decreased by 25 µM SA treatment as compared with HR group (Figure 2A). Similarly, the ratio of Hoechst-positive cell (apoptosis cell) in SA treatment group was also remarkably reduced as compared with HR group (Figure 2B). These results suggested that SA pretreatment inhibited H/R-induced cardiomyocytes apoptosis.

### SA pretreatment repressed mitochondrial apoptosis pathway

**Figure 3:**
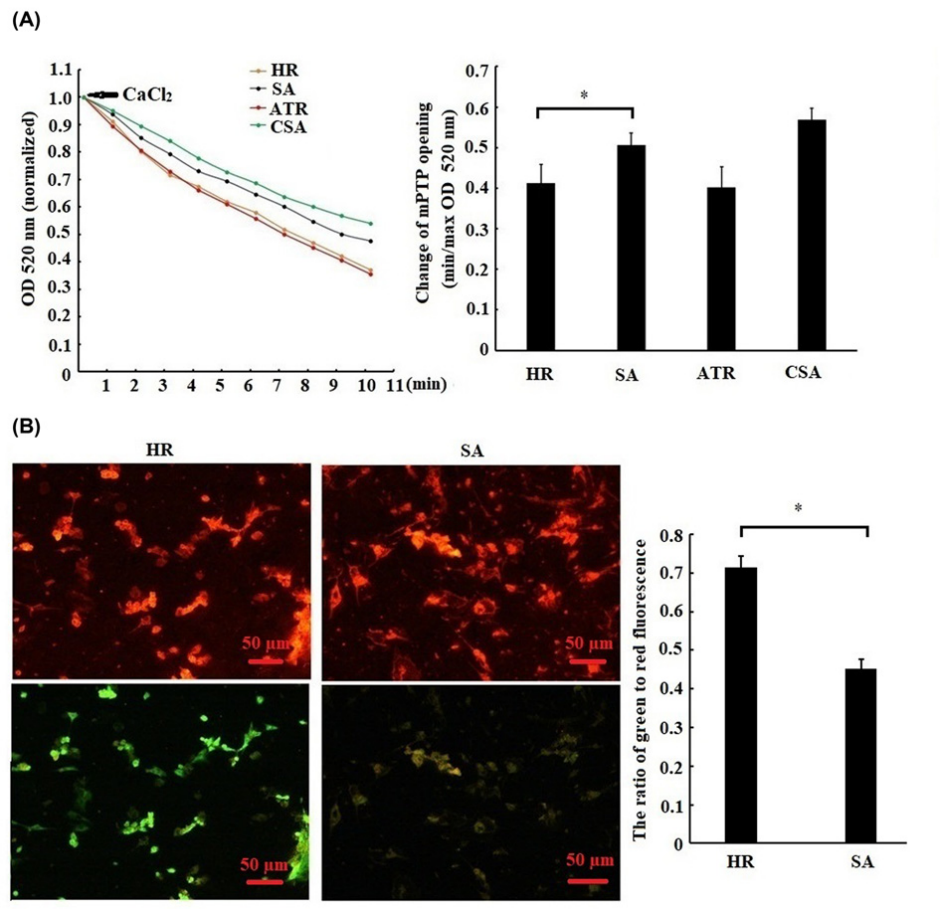
Sappanone A (SA) pretreatment repressed mPTP opening and release of mitochondrial transmembrane potential (Δ*Ψ*m) Mitochondrial apoptosis pathway. H9c2 cardiomyocytes were treated by 25 µM SA for 1 h, followed by 6 h of hypoxia/3 h of reoxygenation. (**A**) mPTP opening was induced by CaCl_2_. Minimum optical density (min OD) represents the OD value recorded at the onset of the experiment (0 min); maximum optical density (max OD) represents the OD value recorded at the end of the experiment (10 min). Min/max OD is negatively associated with the extent of mPTP opening. CSA, cardiomyocytes treated with 0.2 mM cyclosporin a (a mPTP opening inhibitor) was used as a positive control. ATR, cardiomyocytes treated with 20 μM atractyloside (a mPTP opener) was used as a positive control. (**B**) Measurement of the level of Δ*Ψ*m. The level of Δ*Ψ*m was quantified by the ratio of green-to-red fluorescence intensity. Magnification: ×400, scale bars: 50 μm. Data are presented as the mean ± standard deviation from three independent experiments; * *P* < 0.05.

**Figure 4:**
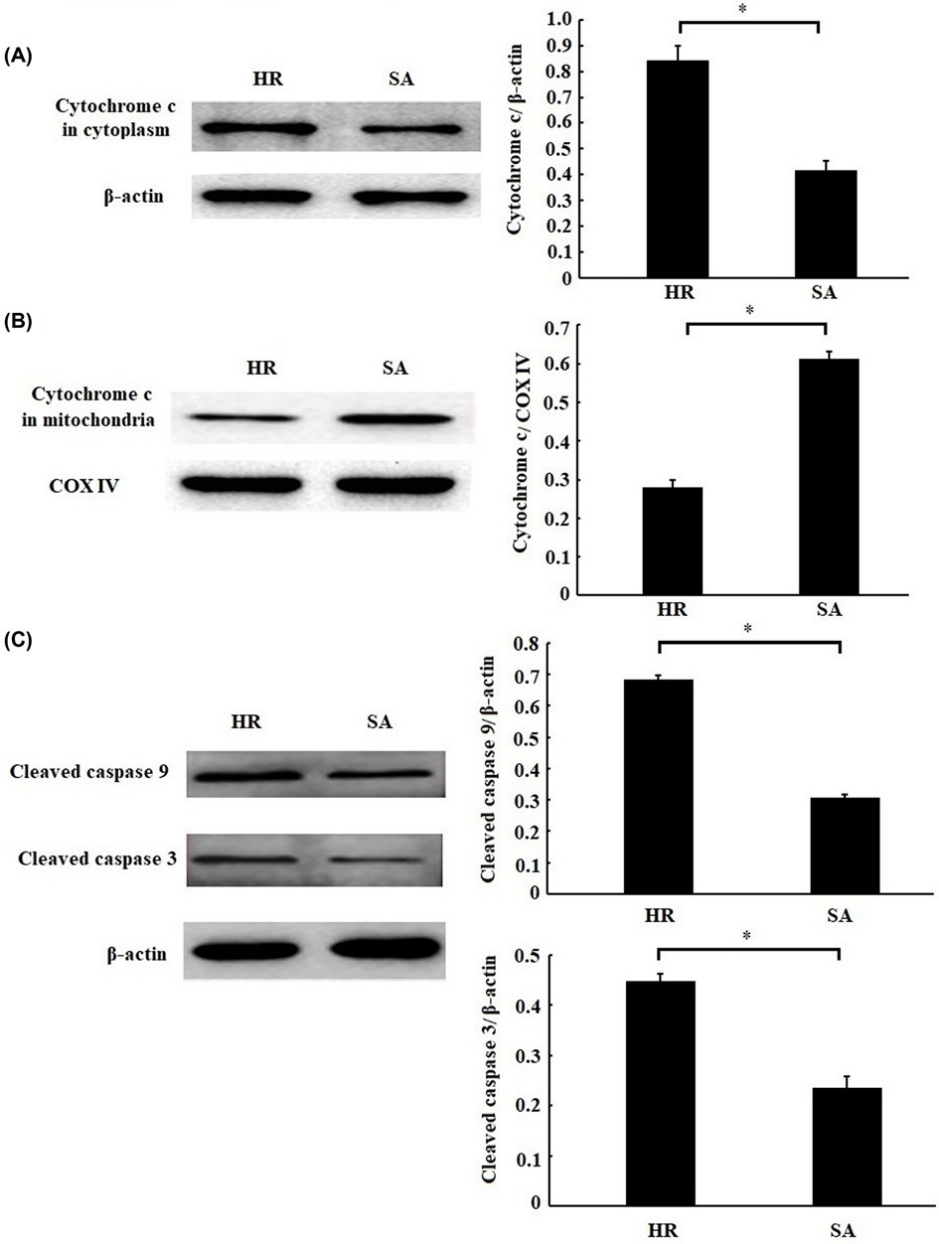
Sappanone A (SA) pretreatment inhibited mitochondrial apoptosis related proteins H9c2 cardiomyocytes were treated by 25 µM SA for 1 h, followed by 6 h of hypoxia/3 h of reoxygenation. The protein expression of cytochrome *c* in cytoplasm (**A**) or mitochondria (**B**) and the cleaved caspase-9 and caspase-3 (**C**) were detected by Western blotting. Data are presented as the mean ± standard deviation from three independent experiments; * *P* < 0.05.

To further confirm whether mitochondrial apoptosis pathway is involved with the inhibition of apoptosis by SA, the changes of mPTP opening, Δ*Ψ*m and mitochondrial apoptosis-related proteins were evaluated. As shown in Figure 3A,B, the mPTP opening and Δ*Ψ*m were remarkably inhibited by SA treatment. Meanwhile, SA treatment prevented the cytochrome *c* releasing from mitochondria into cytoplasm (Figure 4A,B), thereby repressing the cleavage of caspase-9 and caspase-3 (Figure 4C). Taken together, these results suggested that SA pretreatment led to the inhibition of mitochondrial apoptosis pathway.

### SA pretreatment prevented H/R-induced cardiomyocytes injury through PI3K/Akt/Gsk-3β signal pathway

**Figure 5:**
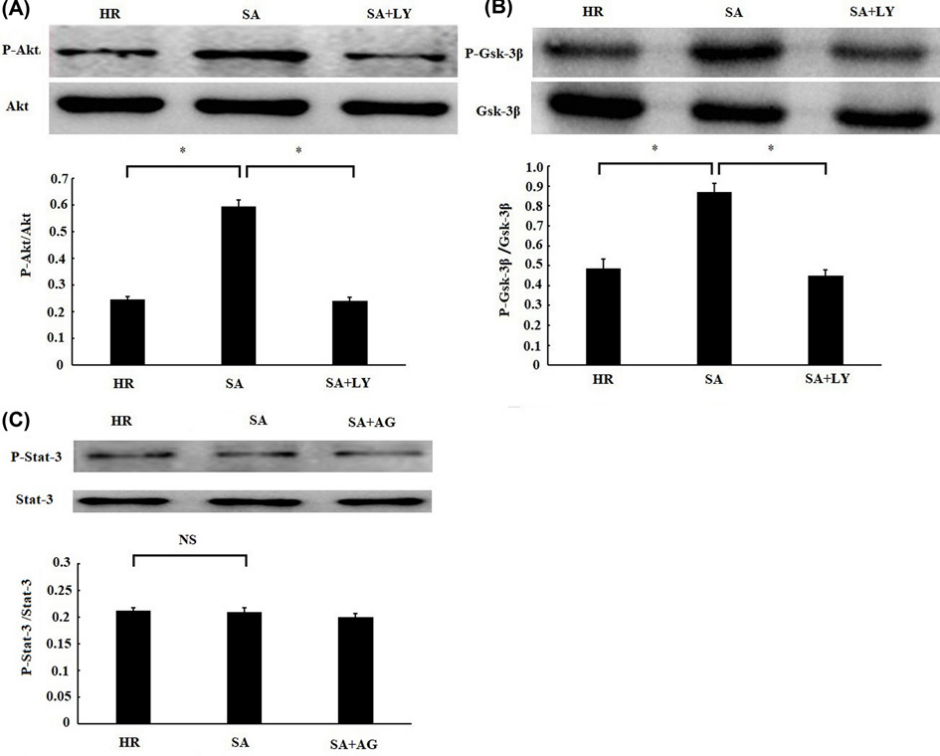
Sappanone A (SA) pretreatment activated PI3K–Akt–Gsk-3β signal pathway H9c2 cardiomyocytes were treated by 25 µM SA for 1 h, followed by 6 h of hypoxia/3 h of reoxygenation. About 10 μM LY294002 (LY), a PI3K inhibitor, was given 1 h prior to SA treatment. About 10 μM AG490 (AG), a STAT3 inhibitor, was given 1 h before SA treatment. The phosphorylation levels of Akt (**A**), Gsk-3β (**B**), and Stat-3 (**C**) were detected by Western blotting. Data are presented as the mean ± standard deviation from three independent experiments. * *P* < 0.05, NS, no significance.

**Figure 6:**
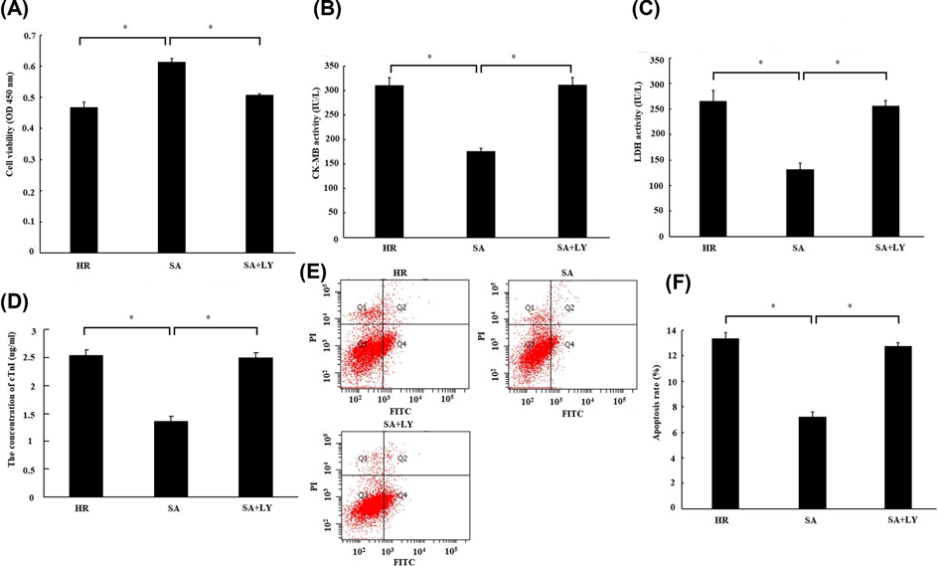
The cardioprotection of Sappanone A (SA) was abrogated by inhibition of PI3K/Akt H9c2 cardiomyocytes were treated by 25 µM SA for 1 h, followed by 6 h of hypoxia/3 h of reoxygenation. About 10 μM LY294002 (LY), a PI3K inhibitor, was given 1 h prior to SA treatment. (**A**) Cell viability was detected by CCK-8 assay. (**B**) The creatine kinase-MB (CK-MB) and (**C**) lactate dehydrogenase (LDH) activity in culture medium were measured by spectrophotometry. (**D**) The concentration of Troponin (cTnI) in culture medium was measured by spectrophotometry. (**E**) Cell apoptosis was detected by flow cytometry. Cells in the lower right quadrant represent apoptosis cells. (**F**) Statistical analysis of apootosis rate. Data are presented as the mean ± standard deviation from three independent experiments; * *P* < 0.05.

To investigate the regulation of PI3K–Akt–Gsk-3β and SAFE signal pathway on the cardioprotection of SA, we measured the changes in the phosphorylation levels of Akt, Gsk-3β and Stat-3 by Western blotting. As indicated in Figure 5A,B, SA pretreatment significantly increased the phosphorylation levels of Akt and Gsk-3β in cardiomyocytes, while the enhancement of their phosphorylation level were abrogated by PI3K inhibitor (LY294002). However, no significant change was found in the level of Stat-3 phosphorlation by SA pretreatment (Figure 5C), which suggested that SA has no effect on SAFE signal pathway. Furthermore, the effect of LY294002 on the cardioprotection of SA was also detected. The protective effects of SA were abolished by LY294002 as evidence that the beneficial effects of SA on cell viability (Figure 6A), myocardial enzymes (Figure 6B,C), the concetration of cTnI (Figure 6D), and cell apoptosis (Figure 6E,F) were compromised by LY294002 treatment. Taken together, these results suggested that SA pretreatment prevented H/R-induced cardiomyocytes injury through PI3K–Akt–Gsk-3β signal pathway.

## Discussion

The dried heartwood of *Caesalpinia sappan L*. is a traditional herb that could be used to accelerate blood circulation and remove blood stasis [11]. Recent studies have reported that the ethanolic extract of *Caesalpinia sappan L*. heartwood could protect HEI-OC1 cells against tert-butylhydroperoxide (t-BHP)-induced oxidative injury [12] and prevent cerebral ischemia reperfusion injury in a rat model [13]. SA is an active constituent isolated from *Caesalpinia sappan L*. heartwood and exhibits antioxidant, anti-inflammatory and anti-apoptotic activities. Thus, we inferred that SA could exert protective effect on MIRI. As expected, our results demonstrated that SA attenuated H/R-induced cardiomyocytes injury in a dose-dependent manner *in vitro.* To our knowledge, this is the first study reporting about the protective effect of SA on H/R-induced cardiomyocytes injury. Our findings suggested that SA may have a potential use for the prevention of MIRI.

The apoptosis pathway is aberrantly activated and excessive apoptosis occurs in the condition of MIRI [14]. Several experiment studies have demonstrated that MIRI is ameliorated through inhibition of apoptosis [15–17]. In the present study, we found that SA treatment significantly inhibited H/R-induced apoptosis; meanwhile, SA treatment inhibited mPTP opening and Δ*Ψ*m release, prevented cytochrome *c* releasing into cytoplasm, and repressed the cleavage of caspase-9 and caspase-3. Similarly, Kang et al. also reported that SA treatment decreased the expression of cleaved caspase-9 and caspase-3 in hypoxia-treated PC-12 cells [8]. Therefore, we inferred that the protective effect of SA on MIRI may partly ascribe to the inhibition of mitochondrial apoptosis pathway.

Growing evidence has demonstrated that active Akt promotes survival of cardiomyocytes *in vitro* and protects against MIRI [18]. Gsk-3β, a crucial downstream molecule of PI3K/Akt signal pathway, typically regulated mPTP opening by PI3K/Akt-mediated phosphorylation [19]. Several studies have reported that ischemic preconditioning [20] or postconditioning [21] could prevent MIRI through activation of PI3K–Akt–Gsk-3β pathway and subsequent inhibition of mPTP opening. Moreover, some drugs, such as rosuvastatin [22] and isoflurane [23], also exerted their protective effects on MIRI via activation of PI3K–Akt–Gsk-3β pathway. In the present study, we demonstrated that SA prevented H/R-induced cardiomyocytes injury through PI3K–Akt–Gsk-3β signal pathway by the fact that SA enhanced the phosphorylation levels of Akt and Gsk-3β, meanwhile the beneficial effect of SA on MIRI was reversed by PI3K inhibitor. These results were generally in agreement with Kang et al. finding that SA increased the phosphorylation levels of PI3K and Akt in PC-12 cells [8]. However, Choo et al. reported the opposite that SA inhibited the activation of the Akt–Gsk-3β signal pathway in receptor activator of nuclear factor-kB ligand (RANKL)-induced osteoclastogenesis in mouse bone marrow macrophages [24]. We speculated that this discrepancy may be ascribed to the difference in cell type and pathophysiological process.

We must acknowledge that there existed some shortages in this present study. First, besides anti-apoptotic activity, SA also possessed antioxidant and anti-inflammatory activities based on previous reports [6,7]. However, we did not provide data on its antioxidant and anti-inflammatory effects on MIRI in the present study. Second, we mainly focus on the change in mitochondrial apoptosis pathway by SA treatment, but the effect of SA on death receptor-mediated apoptosis pathway is not detected. Considering that SA has an inhibition of TNF-α production [6], whether SA has an impact on death receptor-mediated apoptosis pathway should be further evaluated. Finally, we only demonstrated the cardioprotection of SA *in vitro*, the protective effect of SA on MIRI should be confirmed in future studies.

In conclusion, our results demonstrate that SA could prevent H/R-induced cardiomyocytes injury through inhibition of mitochondrial apoptosis and activation of PI3K–Akt–Gsk-3β pathway. SA has a potential use for the prevention of MIRI.

## Abbreviations

H/R: hypoxia/reoxygenation
MIRI: myocardial ischemia reperfusion injury
mPTP: mitochondrial permeability transition pore
SA: Sappanone A
